# A non-synonymous single-nucleotide polymorphism in the gene encoding Toll-like Receptor 3 (TLR3) is associated with sero-negative Rheumatoid Arthritis (RA) in a Danish population

**DOI:** 10.1186/1756-0500-7-716

**Published:** 2014-10-10

**Authors:** Magdalena J Laska, Bettina Hansen, Anne Troldborg, Tove Lorenzen, Kristian Stengaard-Pedersen, Peter Junker, Bjørn A Nexø, Hanne M Lindegaard

**Affiliations:** Department of Biomedicine, Aarhus University, Bartholin Building 1240, Wilhelm Meyers Alle 4, 8000 Aarhus C, Denmark; Department of Rheumatology, Odense University Hospital, 5000 Odense, Denmark; Department of Rheumatology, Aarhus University Hospital, 8000 Aarhus C, Denmark; Department of Rheumatology, Vejle Hospital, 7100 Vejle, Denmark

**Keywords:** Rheumatoid arthritis, Genetic predisposition to disease, Autoimmune diseases, Polymorphism

## Abstract

**Background:**

It has been suggested that polymorphisms in Toll-like Receptors (TLRs) are associated with Rheumatoid Arthritis (RA), but the implicated alleles have differed between studies. The aim of this investigation was to explore whether polymorphisms of TLR genes are associated with RA in a predominantly Caucasian population from Denmark using a case–control approach.

**Findings:**

DNA samples (3 university hospital outpatient clinics) were obtained from patients with RA (n = 704) and healthy controls (n = 639) in a Danish population. TLR single nucleotide polymorphisms (SNPs) were selected based on the previously reported associations with chronic autoimmune diseases. Genotyping for the TLR SNPs was performed using Sequenom Multiplex technology.

We identified one SNP in TLR3, [(rs3775291, *P* = 0.02, OR (95% CI) 1.31 (1.1087-1.5493)] significantly associated with the whole RA cohort. Subgroup analysis according to IgM rheumatoid factor (RF) and anti-cyclic citrinullated peptide (CCP) status suggested a significant association of sero-negative RA with the rs3775291 A allele and disease activity in this subset.

**Conclusion:**

These observations on a RA population of Danish ancestry suggest that variations in the TLR3 locus may be implicated in the pathogenesis of sero-negative RA. Since this TLR3 SNP has previously been associated with systemic lupus erythematous (SLE), the present findings support the notion that TLR3 genetic variants may represent a common risk factor in different chronic inflammatory conditions, including RA and SLE.

## Findings

Rheumatoid arthritis (RA) is a chronic, systemic and inflammatory joint disease that leads to bone and cartilage destruction, as well as a wide variety of extra-articular manifestations [1]. The causes of RA are largely unknown, however, the role of genetic factors is evident, with the major histocompatibility complex (MHC) region as major contributor [2]. In spite of that, this association accounts for only one-third of the genetic susceptibility, as non-Human Leukocyte Antigen (HLA) genes are also known to be involved [3].

Toll-like receptors (TLRs) are involved in the detection of environmental signals and the regulation of dendritic cell (DC) function and are therefore the subject of investigations in autoimmunity. TLRs are expressed both by immune cells and by resident cells of the joint, including fibroblast-like synoviocytes (FLS), which in RA play a key role by producing cytokines that perpetuate inflammation and proteases that contribute to cartilage destruction [4].

Several studies have examined the potential pathogenetic contribution by TLR polymorphisms to the manifestations of RA, but the results have been conflicting [5–7]. We have recently reported that polymorphisms within TLRs are associated with Systemic Lupus Erythematous (SLE) in a Danish lupus cohort [8]. Here, we hypothesized that some of the SLE associated markers might likewise be associated with RA. To test this, we investigated potential associations between polymorphisms within TLR genes and RA phenotype and severity.

## Materials and methods

### Ascertainment of patients

704 RA patients were randomly recruited from 3 university hospital outpatient clinics. All patients fulfilled the American College of Rheumatology 1987 revised criteria for RA [9]. 639 controls were healthy individuals without RA, autoimmune or systemic disorders nor a family history of RA. The study was approved by the regional ethics committees of Southern and Mid Denmark (no S20090051 and 1-10-72-388-12). Informed and written consent was provided by all the participants.

### Characterization of disease activity and outcomes in patients

Health Assessment Questionnaire (HAQ score, 0 to 3), Visual Analogue Scale (0 to 10) (VAS pain, global and doctor) and Disease Activity Score in 28 joints (DAS28 scale, 0 to 10) were calculated. Anti-CCP (IgG antibodies) was determined by a second-generation ELISA with 25 μg/ml as the cut-off point. Serum C-reactive protein (CRP; mg/ml) and erythrocyte sedimentation rate (mm/h) were measured using standard laboratory methods. IgM-RF was detected by ELISA; the cut-off level was >16 IU/ml (~95th percentile of healthy subjects).

### Nucleic acid extraction

Genomic DNA was isolated from peripheral blood samples using a standard method, which included separation and lysis of the nuclear cells, treatment with proteinase K, extraction with phenol-chloroform and ethanol precipitation. DNA concentrations were determined using a Quanti-iT™ PicoGreen® ds DNA kit (Life Technologies Europe BV, DK).

### Genotyping

SNPs selected for the assays were primarily functional, and previously reported to be associated with SLE or other autoimmune disease [8]. Genotypes of 22 TLR SNPs were determined by Sequenom MassArray technology (Sequenom iPLEX assay, San Diego, USA), in accordance with the manufacturer’s instructions. The reactions were analyzed by MALDITOF mass spectrometry on the Sequenom equipment, and the results were analyzed using the MassARRAY Typer 4.0. The SNPs and primers sequenced were published previously [8].

### Statistical analysis

Frequencies of the TLR genotypes were tested for Hardy-Weinberg equilibrium among the controls using the standard goodness-of-fit test. Similarity of genotype and allele distribution between patients and controls was tested with chi-square tests for 3 × 2 contingency tables. Differences in the disease characteristics between patients were analyzed using Student’s t test or Mann–Whitney U test. Corrections for false rate discovery were applied using Benjamin and Hochberg formula. Power analysis revealed that with 704 patients and 639 controls we were able to demonstrate a 1.6 fold increase in frequency of the particular genotypes in RA patients compared with controls ( power 95%, ɑ = 0.05).

## Results

### Association of TLR SNPs with RA in a Danish population

Of 22 polymorphisms investigated, only rs3775291 located in TLR3, showed significant allelic association with RA in this Danish cohort. The carrier frequency of the rs3775291A allele was significantly increased in RA patients compared with healthy controls (p(A)_cases_ =0.32 vs p(A) _controls_ =0.26) [*P* =0.02, OR (95% CI) 1.31 (1.1087-1.5493)]. The genotype frequencies are given in the supplemental data, Table 1. The TLR3 marker was not associated with age at inclusion, neither in the RF-negative nor the total groups. Nor was there any effect on age at onset. For the remaining TLR SNPs no significant associations were observed between the genotypes or allele frequencies and the presence of RA. However, several other TLRs showed weaker signs of SNP association.

**Table 1:** **Genotype frequencies for rs3775291in relation to RA**

Crosstab					
Count					
		TLR3_rs3775291			Total
		AA	GA	GG	
State	Case	66	319	319	704
	Cntl	49	240	350	639
Total		115	559	669	1343

### Association of TLR3 rs3775291 and clinical parameters

To analyze the relation of rs3775291 to disease manifestations we recoded the SNP into the numbers of A-alleles present in the genotype, i.e. AA = 2; AG = 1 and GG = 0, corresponding to co-dominance of the investigated marker. Subsequently, we used regression analysis to calculate the regression coefficient to the numbers of A’s, thereby enabling us to evaluate the relevance of the marker. Stratification according to IgM-RF among the cases revealed no evidence of different associations between the compared subgroups [(coef_TLR_ =0.034 (*P* = 0.63)].

**Figure 1:**
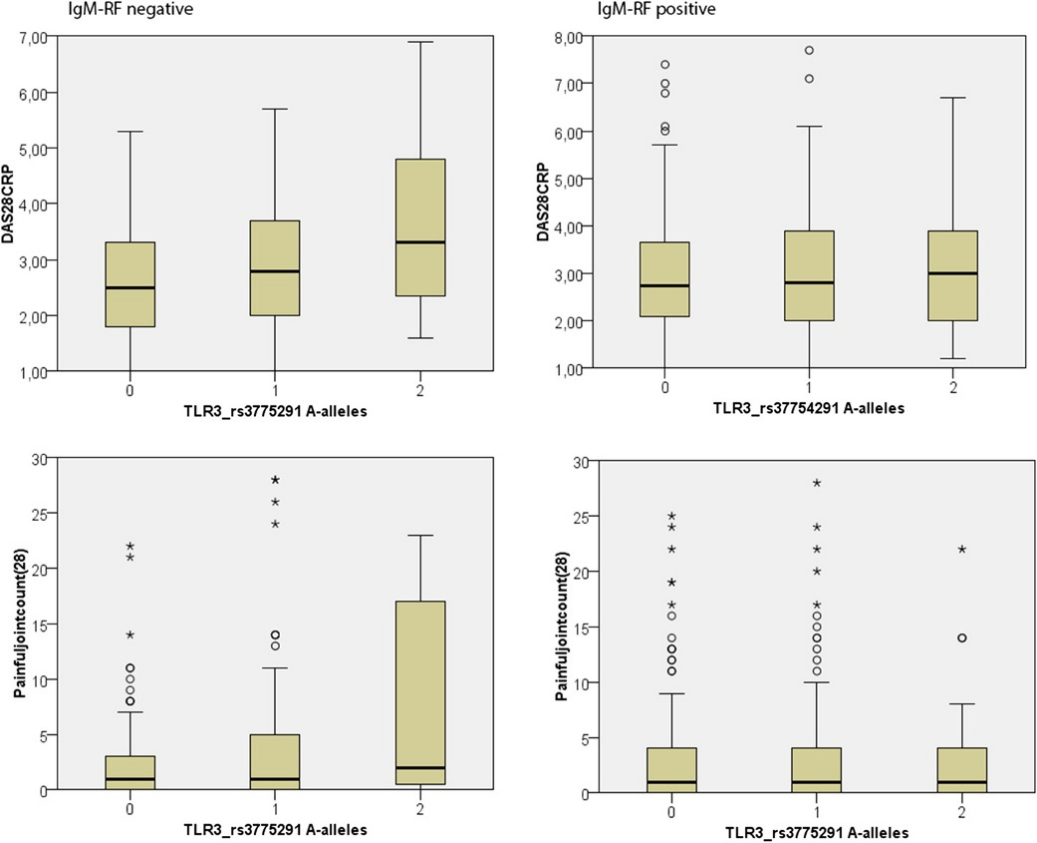
**Relation of disease activity scores, as measured by DAS28, to the TLR rs3775291 in the two subpopulations.** The box plot with median marked with black line, and hinges indicating 25th and 75th percentile. Whiskers represent extreme values within 1.5 times the interquartile range. The circles represent outliers and the asterisks are extreme outliers.

Next, we stratified the patients by IgM-RF status and calculated the remaining clinical parameters accordingly (Table 2). Even though this stratification reduced the number of subjects eligible for the analysis, subgroups maintained numerosity comparable with the entire study cohort. Notably, three parameters (swollen join count 28, painful joint count 28, DAS28CRP), relating to active joint disease in RA were associated with the TLR3 marker among RF-negative, but not among RF-positive patients. The results remained significant after Bonferroni correction for the multiplicity of testing. RF-positive RA occurred significantly earlier among women than men (onset =47 vs 50 years, p =0.006). RF-negative RA had similar onset in both genders (55 years). There was a non-significant tendency that onset occurred later in homozygote rs3775291AAs than in heterozygote AGs among the RF-negative (onset =60 vs. 54 years, p =0.17). A similar tendency was present in the RF-positive (onset =51 vs. 48 years, p =0.19).Figure 1 presents the relation of disease activity score, as measured by DAS28, to the TLR3 rs3775291 in the two subpopulations. The figure also shows the relation of the number of painful joints to the marker. Similar results were obtained when patients were segregated into anti-CCP positive and anti-CCP negative RA.

**Table 2:** **Association of various clinical RA markers with TLR3 rs3775291**

	RF negative		RF positive	
Parameter	coef_TLR_	p-coef	coef_TLR_	p-coef
spd	0.032	0.689	0.088	0.057
Anti-CCP	0.075	0.355	−0.004	0.939
Swollenjointcount(28)	0.159	0.048	−0.016	0.724
Painfuljointcount(28)	0.274	0.0006	−0.017	0.717
CRP(mg/l)	−0.030	0.713	0.006	0.604
VASpatientglobal	0.099	0.222	0.006	0.984
DAS28CRP	0.245	0.002	0.014	0.760
HAQscore	0.014	0.989	−0.030	0.514

Spd, surfactant protein D (serum); Anti-CCP, anti-cyclic citrullinated peptide antibodies; Swollenjointcount(28), swollen joint count (maximum 28 joints); Painfuljointcount(28) tender joint count (max 28 joints); CRP, c-reactive protein (mg/l); VASpatientglobal, Visual Analogue Scale; DAS28CRP, composite measurement Disease acitivity 28 joint crp; HAQscore, Health Assessment Questionnaire (HAQ score, range 0-3).

## Discussion

A variety of experimental models have suggested a role for TLRs in the pathogenesis of RA [10]. The present data provide evidence for an association between the TLR3 gene variant (rs3775291) and RA. In addition, disease activity measures including DAS28 and swollen joint counts were associated with rs3775291 in sero-negative RA. This does not necessarily mean that the rs3775291 has no influence in sero-positive RA patients, but any effect may be masked by the greater influence of sero-positivity.

The results of the present study support the notion that the genetic background may differ between IgM sero-positive vs- sero-negative RA thereby contributing to the concept of rheumatoid arthritis as 2 separate and distinctive disease entities [11]. For example, previous studies have suggested that sero-positive and sero-negative RA have distinct MHC class II cell surface receptor (HLA-DR) associations, especially in disease of early onset, in addition to well established clinical differences [3]. Although the functional consequences of genetic variability in the TLR3 locus have not been defined in detail it is tempting to speculate that polymorphisms in the TLR3 gene may not only have an impact on the risk of developing sero-negative RA but may also play a role as disease activity modifier in sero-negative RA as reflected by the association with DAS28 and swollen joint count.

The SNPs analyzed in this study were selected based on previous associations with autoimmune diseases and tagging properties, and are contained within TLR sequences. Some TLRs polymorphism have been shown to be associated with susceptibility and the clinical profile of RA, but few studies have been conducted in the past on each of these associations [5, 12]. To the best of our knowledge, this is the first study demonstrating an association between the TLR3 polymorphism rs3775291 and disease activity in RA. Of note, rs3775291 was included as a candidate marker in genome-wide association studies (GWAS) on RA in a Spanish population, but the analysis did not reveal a genome-wide significance level [13]. This outcome could be explained if much of the missing genetic control in RA is due to gene variants that are too rare to be picked up by GWAS studies and have relatively large effects on risk.

The rs3775291 A allele has previously been associated with female SLE in a Danish population and with sub-acute sclerosing panencephalitis in a Japanese study [8, 14]. Moreover, genetic studies have provided evidence concerning the role of rs3775291, Leu412Phe with respect to the susceptibility to viral infections [15]. Thus, although the functional consequences of the rs3775291 A polymorphism in RF sero-negative RA have not been settled, our findings may lend support to an infectious trigger in sero-negative rheumatoid arthritis.

In summary, this study provides evidence that the TLR3 rs3775291 locus associates with the occurrence and disease activity in sero-negative RA. This and previous findings in SLE patients [8] suggests that TLR3 genetic variants may represent shared pathogenetic pathways in chronic inflammatory conditions including RA and SLE.
